# Cage size, movement in and out of housing during daily care, and other environmental and population health risk factors for feline upper respiratory disease in nine North American animal shelters

**DOI:** 10.1371/journal.pone.0190140

**Published:** 2018-01-02

**Authors:** Denae C. Wagner, Philip H. Kass, Kate F. Hurley

**Affiliations:** 1 Koret Shelter Medicine Program, University of California at Davis, Davis, California, United States of America; 2 Department of Population Health and Reproduction, University of California at Davis, Davis, California, United States of America; University of Minnesota, UNITED STATES

## Abstract

Upper respiratory infection (URI) is not an inevitable consequence of sheltering homeless cats. This study documents variation in risk of URI between nine North American shelters; determines whether this reflects variation in pathogen frequency on intake or differences in transmission and expression of disease; and identifies modifiable environmental and group health factors linked to risk for URI. This study demonstrated that although periodic introduction of pathogens into shelter populations may be inevitable, disease resulting from those pathogens is not. Housing and care of cats, particularly during their first week of stay in an animal shelter environment, significantly affects the rate of upper respiratory infection.

## Introduction

Feline upper respiratory infection (URI) has been described as one of the most important causes of morbidity and mortality for cats in North American animal shelters [1]. In addition to being a common reason for euthanasia[2], URI can have acute and chronic sequelae for feline health. Even at those shelters that have the wherewithal for treatment, caring for sick animals consumes scarce shelter resources. With reported incidence as high as 30% [2, 3], these costs may be substantial. In this context, prevention of disease is critical; yet, in spite of significant investment in improved shelter facilities and extensive efforts at management [4], feline URI remains one of the most significant disease concerns for shelter managers [5].

Management of URI is complicated by the multi-factorial nature of this condition in cats. The pathogens implicated in feline URI include feline herpesvirus (FHV-1) and feline calicivirus (FCV) as the most common primary causes, as well as *Mycoplasma spp*., *Chlamydophila felis*, and *Bordetella bronchiseptica* [6–10]. The frequent carrier state for many of these pathogens means that even apparently healthy cats are likely to enter shelters subclinically infected and subject to recrudescence of latent infections[11–14]. In particular, recrudescence of FHV-1 has been specifically linked to stress, and multiple studies have documented an association between stress and URI in shelter cats [15–17]. Thus, prevention of pathogen transmission and support of behavioral health are both essential to limit URI in shelter cats, but must be balanced with one another. Rigorous isolation prevents exposure but may create stress for cats, while enriched environments and social interaction may reduce stress but increase disease transmission. A successful strategy to mitigate shelter URI must serve these two potentially conflicting goals while simultaneously preparing and presenting cats for adoption—a significant challenge in the resource limited environment of many shelters.

Studies of URI in shelter cats have tended to focus on management strategies at the individual cat versus population level. Multiple studies have evaluated the success of various antibiotics for empirical treatment of URI in pet cats and shelter cats.

Preventive strategies directed at individual cats have also been evaluated within shelters, including supplementation with lysine and vaccination for FHV-1 and FCV [18, 19]. No preventive benefit was found for lysine supplementation, while at best modest benefits have been reported for vaccination [19–21].

Behavioral as well as medical interventions have been investigated. Consistent human interaction tailored to the cat’s temperament was significantly associated with increased secretory immunoglobulin A, reduced shedding of URI pathogens and lower risk of clinical URI [3, 22, 23]. However, the overall incidence of URI in cats in the enrichment study was still 34%, comparable to levels reported in other shelters without an equivalent enrichment program [2, 14, 21]. This suggests that other preventive measures, potentially applied at a population rather than individual level, may be required in combination with individually-directed methods to prevent this common condition.

Variation in risk for feline URI has been specifically linked to environmental factors at the household or cattery level, including level of hygiene, number of cats, and household type [9, 24]. A comparison of data between shelters also suggests the importance of population-level factors. Studies have documented frequency of URI in United States shelters from 19% [19] to ~ 30% [2, 3, 21] (as a percent of intake). By contrast, in a study of five shelters in the United Kingdom, URI frequency as a percent of intake was only 4% overall, with a range at individual shelters from 2% to 14%.

In addition to environmental and host risk factors, the role of pathogen carriage and transmission must be considered. Previous studies confirm that potential exists for rapid and efficient disease expression and transmission in shelters and shelter-like environments. [6, 11, 14–16]. Carriage of pathogens, particularly FHV-1 and *Bordetella bronchiseptica*, is significantly linked to URI risk in cats within a shelter[14]. However, it is unknown to what extent differences in URI frequency between shelters are linked to differences in the frequency of pathogen carriage in newly admitted cats, versus differential expression and transmission of pathogens driven by environmental, management and host factors. In calves with bovine respiratory disease, a similarly multi-factorial respiratory disease, the mix of pathogens associated with disease tends to be similar between populations, but severity and frequency of pathogen expression is determined by husbandry and environmental factors (such as weaning, transportation, and weather) [25].

The present study aimed to document variation in risk of URI between North American shelters; determine whether this reflects variation in pathogen frequency on intake or differences in transmission and expression of disease; and identify modifiable environmental and population health factors linked to risk for URI.

## Materials and methods

### Ethics statement

This study protocol was reviewed and approved by the University of California—Davis Institutional Animal Care and Use Committee (Approval #13000). Permission for participation of shelter cats in this study was obtained from the managing veterinarian at each shelter.

### Shelters

Nine shelters participated in the study. Shelter enrollment in the study was convenience-based and criteria for inclusion in the study were a willingness to participate; presence of a veterinarian to serve as lead contact for the study; existence of a defined set of criteria for URI recognition at the shelter; willingness to comply with study protocol; ability to collect required data daily, and completion of an online survey. The study was conducted from August 1, 2008 through July 31, 2009.

### URI case definition

Each shelter that was enrolled had an on-site veterinarian and defined set of criteria for URI recognition. A universal case definition for feline URI across all participating shelters was not implemented. Case definitions ranged from very broad—any symptoms of upper respiratory disease present—to more specific, where several symptoms needed to be present prior to diagnosis. Case definitions included some degree of sneezing, along with ocular and/or nasal discharge. Individual shelter URI case definitions are available in the supporting information S1 Doc. Shelter URI Criteria.

### Shelter baseline data

Prior to the start of the study, shelters completed a 47-question online survey (S1 Survey link) to establish shelter baselines for housing, management and environmental factors (summarized in Table 1). Specific questions were asked about housing during the first 7 days of shelter care, referred to as “intake housing”. Monthly updates were received via email from each shelter’s veterinarian to report any major changes from the baseline data provided in the survey for housing, management, or the environment that occurred in the shelter during the 12-month study period.

**Table 1:** Baseline housing and environmental factors.

Shelter	Intake cage floor space ^a^	Hiding area provided in cages during the first week of stay	# Cage moves during the first week of stay	Mixed ages (juveniles and adults) in intake rooms	Vaccination intranasal modified live FVRC^b^ at intake
1	2	Sometimes	≤2	Yes	Yes
2	1	Sometimes	≤2	Yes	Yes
3	1 and 3	No	>2	No	No
4	1	Sometimes	>2	Yes	No
5	1	No	>2	No	Yes
6	1	Yes	>2	Yes	No
7	1 and 2	No	>2	Yes	No
8	3	Yes	≤2	No	Yes
9	1	Yes	>2	Yes	No

^a^1 = 3 to <6 ft^2^, 2 = 6–8 ft^2^, 3 = >8–10 ft^2^ (0.28–0.56m^2^, 0.56–0.74m^2^, > 0.74–0.93m^2^)

^b^Feline viral rhinotracheitis (feline herpesvirus-1) and calicivirus

### Shelter data collection

All shelters used an online URI database (described below) where shelter staff recorded the daily number of adult cats in the shelter population categorized by health status (with or without URI), and each day’s intake of adult felines (new cats entering the shelter population). Feral cats were not included in this study. Cats were observed daily and recorded as either not sick with URI (and therefore at risk for URI) or sick with URI. For each new case of URI observed, the following data were recorded in the online URI database: Cat ID#, intake date and URI diagnosis date. Shelters also had the option to enter outcome date and outcome type (e.g. adoption versus euthanasia), but this was not required nor consistently used and therefore not included in the analysis.

The online URI database was developed by the Computing and Technology Services (CATS), UC Davis School of Veterinary Medicine, hosted by UC Davis Koret Shelter Medicine Program. Prior to the start of the study, a one-hour internet sharing session (using Breeze Meeting by Adobe, San Jose, California) was held for each shelter to train the shelter veterinarian and staff on the use of the online database and to answer questions. Each participating shelter had a unique ID and login password to access the online database. Shelters had access to all their data and could monitor their URI rates throughout the study period. The database provided each shelter with a monthly report that summarized feline URI via a linear graph that displayed the shelter’s URI rate in bimonthly intervals, with the ability to view any time range of interest.

### Shelter URI rate calculation

To determine the monthly URI rate, the total number of new cases of URI was divided by the total number of cat-days at risk for URI (the sum of the daily inventory of cats without URI) for that month. URI rates in this study were reported per 1000 at risk cat days. Cats diagnosed with URI at the time of intake, or on day 1 or day 2 of their shelter stay were excluded from the calculation, as these were considered to be pre-existing cases of URI and not shelter-acquired disease.

### PCR sampling for URI pathogens at intake

PCR sampling was performed at a subset of five shelters during the study period. Selection was based on having skilled medical staff available for sample collection or shelter location proximal to the research team. Cat handling and PCR sampling were consistently performed. Samples were collected by trained RVT and Veterinary staff via the research team (at 4 shelters) or via the shelter medical staff (1 shelter). Approximately 20 cats observed to be healthy at intake were sampled at each shelter during a one-week sampling period every three months. Cats showing any sign of URI, and those designated as feral by shelter staff or that could not be handled for sampling were excluded. Cats were sampled within 24 hours of intake at each of the five shelter facilities. Because sampling was only performed at one shelter at a time, a five-week continuous period was needed to sample cats at the five shelters. (In a few cases, it was not possible to get 20 samples because of low intake during the sampling period.) Samples were collected onto sterile cotton swabs with plastic sticks. Samples for PCR testing consisted of one oropharyngeal swab and one conjunctival swab from each cat. The two sample swabs were placed into a single red top tube and labeled for the individual cat.

Samples were stored in a cooler during each sampling day and then placed in a freezer (-20°F or -29C) until all the samples for that shelter were collected for that sampling period. Samples were submitted (delivered or mailed) to IDEXX Laboratories (Sacramento, California) for real-time quantitative polymerase chain reaction (RT-PCR) testing (test code 2512 RealPCR^™^ FURD panel) designed to detect the following feline upper respiratory disease agents: *Chlamydophila felis*, feline calicivirus (FCV), feline herpesvirus-1 (FHV-1), *Bordetella bronchiseptica* and *Mycoplasma felis*. All assays were designed and validated according to industry standards (Applied Biosystems, User Bulletin #3). Target genes for each application were: *B*. *bronchiseptica*: hemagglutinin fusion protein gene (FhaB), AF140678; *C*. *felis*: outer membrane protein A (OmpA), AP006861; feline herpesvirus 1: glycoprotein B (18), feline calicivirus: ORF 1, AF109465; M. felis: ssr RNA—ITS-1, AF443608. The total number of cats sampled at intake in the five shelters over the one-year period was 329 cats.

### Statistical analysis

Variables were originally obtained reflecting the range of practices considered possible risk factors for URI and which could reasonably be assessed via questionnaire. However, for some variables, all shelters followed the same practices, preventing comparison. For example, all shelters vaccinated cats on intake with a modified live parenteral vaccine for feline calicivirus, herpesvirus and panleukopenia; fed a consistent diet daily from day to day within the shelter (as compared to some shelters where a variety of donated food is fed from day to day); and used a disinfection labeled as effective for all pathogens implicated in feline URI.

The following variables were examined using Poisson regression with robust variance estimation to account for clustering of cats within shelters (Stata 13.1/IC, StataCorp LP, College Station, Texas) for possible associations with monthly URI rates between shelters: double compartment housing (no/yes), intake housing floor space (3–6 ft^2^, 6–8 ft^2^, >8-10ft^2^ {.28-.56m^2^, .56-.74m^2^, >.74-.93m^2^}), hiding space provided in intake housing (no, sometimes, always), mixed-age housing (no, yes), frequency of cat moves in and out of the cage in the first week (≤ 2 moves, >2 moves), use of intranasal vaccine (no, yes) and monthly shelter intake (natural log transformation). A p value < 0.05 was considered significant.

In a separate analysis involving a subset of five shelters, Poisson Regression was used to examine the association of pathogens at intake with monthly URI rate. A p value < 0.05 was considered significant.

## Results

### Shelters and shelter data

Nine North American animal shelters participated in the study. Data was collected and was included in the analysis from August 1, 2008 through July 31, 2009.

### URI summary statistics

In total, 18,373 adult cats in these nine North American shelters were included, with 210,987 cat days at risk and 31,924 URI cat days recorded (Table 2). One shelter had URI incidence rates consistently below 3 cases/1000 cat days at risk and another shelter was below 11 cases/1000 cat days at risk for the full study period. There were four shelters that had variable rates (high and low levels of URI at different times in the year) and there were three shelters with URI incidence rates equal to or above 15 cases/1000 cat days at risk for more than half of the study time. The overall URI rate for the year varied almost 50-fold between shelters, from 0.7 to 33.4 cases/1000 cat days at risk. The overall average annual URI rate for adult cats was 14.8 cases/1000 cat days at risk. Overall, 17% of admitted cats developed URI and the percentage for individual shelters ranged from ~ 3% to > 29%.

**Table 2:** Shelter URI summary statistics.

Shelter	Adult feline intakes	Cat days at risk	URI cat days	Adult URI cases (occurring >2 days in shelter)	Avg. yearly URI rate (URI cases divided by cat days at risk times 1000)
1	3148	31396	1173	187	6.0
2	747	18788	1297	124	6.6
3	1733	20904	854	119	5.7
4	996	13742	2255	183	13.3
5	704	9286	718	88	9.5
6	4137	52231	12324	1223	23.4
7	4214	28353	11208	949	33.5
8	652	29015	263	20	0.7
9	2042	7272	1829	236	32.5
**Totals**	**18373**	**210987**	**31924**	**3129**	**14.8**

**Fig 1:**
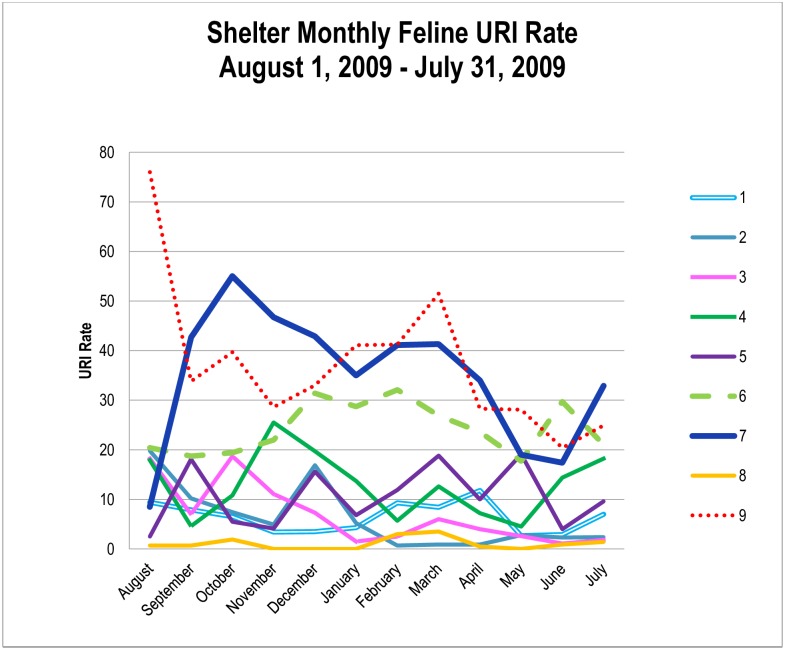
Monthly feline URI rate during the study period.

Monthly email updates were received from all the shelters documenting any changes in management and housing of cats. There were no changes in vaccination type or use and no changes in disinfectant use. Two shelters reported high staff turnover at various points, however no major management changes were recorded in the nine shelters over the study period. Two shelters introduced new larger double-compartment individual cat housing in May of 2009, which was accounted for in the analysis. Monthly data summary is shown in Fig 1.

### Risk factors for URI

The variables that showed highly significant protective effects on adult URI incidence were: Intake housing floor space >8 ft^2^ (.74m^2^) and two or fewer housing moves during the first week of stay. Variables associated with significantly increased URI incidence rates for adult cats were providing hiding spaces (sometimes or always) and use of an intranasal vaccine. Cat intake number (Ln) and mixed-age housing were not significant (Table 3).

**Table 3:** Poisson regression: URI risk factors in adult cats.

Variable	IRR	95% Confidence interval	P value
Cat intake (ln)	1.23	0.75–2.02	0.41
Intake Housing Floor Space <6 ft^2^	1.00		
Intake Housing Floor Space 6 to <8 ft^2^^a^	0.69	0.45–1.06	0.09
Intake Housing Floor Space >8 ft^2^^b^	0.078	0.06–0.10	<0.0001
Hiding Space in Intake Housing (Never)	1.00		
Hiding Space in Intake Housing (Sometimes)	7.29	3.04–17.50	<0.0001
Hiding Space in Intake Housing (Always)	5.98	3.46–10.32	<0.0001
Mixed-Age Housing present	1.00		
No Mixed-Age Housing	1.89	0.78–4.58	0.16
Number of Housing Moves (> 2 in first week of stay)	1.00		
Number of Housing Moves (≤ 2 in first week of stay)	0.19	0.14–0.25	<0.0001
Intranasal Vaccine Use (FVRC^c^)	1.66	1.13–2.44	0.010

^a^0.56–0.74m^2^

^b^ >0.74m^2^

^c^ Feline viral rhinotracheitis (feline herpesvirus-1) and calicivirus

### Intake cat pathogen prevalence and shelter URI incidence rates

Pathogen prevalence is shown in Table 4.

**Table 4:** PCR pathogen prevalence in healthy cats at intake.

Shelter	Cats tested	Intake Cat PCR Positives					Yearly URI Rate (URI cases/1000 at risk days)
		*Chlamydophila felis*	*FCV*^a^	*FHV-1*^b^	*Bordetella bronchiseptica*	*Mycoplasma felis*	
A	60	1.6% (1)	15.0% (9)	35.0% (21)	3.3% (2)	28.3% (17)	32.3
B	80	1.2% (1)	15.0% (12)	23.7% (19)	3.8% (3)	28.7% (23)	31.8
C	68	(0)	22.0% (15)	26.5% (18)	2.9% (2)	38.2% (26)	13.3
D	63	1.6% (1)	11.1% (7)	1.6% (1)	(0)	31.7% (20)	5.7
E	58	(0)	6.9% (4)	36.2% (21)	10.3% (6)	6.9% (4)	0.7

^a^Feline calicivirus

^b^Feline herpesvirus-1

There were no significant effects of the proportion of pathogens present in cats at intake on shelter URI incidence rates (Table 5).

**Table 5:** Pathogen prevalence in cats at intake and shelter URI incidence rate.

Pathogen	IRR	Robust Std. Err	Z	P> | z |	95% Cond. Interval
*Chlamydophila felis*	3.676908 (exposure)	28.22569	0.17	0.865	1.07e-06–1.26e+07
Feline calicivirus (FCV)	1.956754 (exposure)	3.788967	0.35	0.729	0.0439855–87.04884
Feline herpesvirus-1 (FHV-1)	4.188334 (exposure)	3.876766	1.55	0.122	0.6825907–25.69936
*Bordetella bronchiseptica*	.3133567 (exposure)	0.6313917	-0.58	0.565	0.0060385–16.2
*Mycoplasma felis*	1.415787 (exposure)	2.994384	0.16	0.869	0.0224238–89.38946

## Discussion

This study demonstrated that, although periodic introduction of pathogens into shelter populations may be inevitable, disease resulting from those pathogens is not. There was wide variation in URI rate between shelters in association with modifiable environmental risk factors and independent of the frequency of pathogen carriage in cats at intake. Variations in URI rate are expected to reflect variable recrudescence of latent FHV-1 infection as a result of stress, in addition to transmission of new infections. Thus, a lower URI rate may be a marker for improved behavioral well-being for cats as well as better health, lending increased importance to these findings.

**Fig 2:**
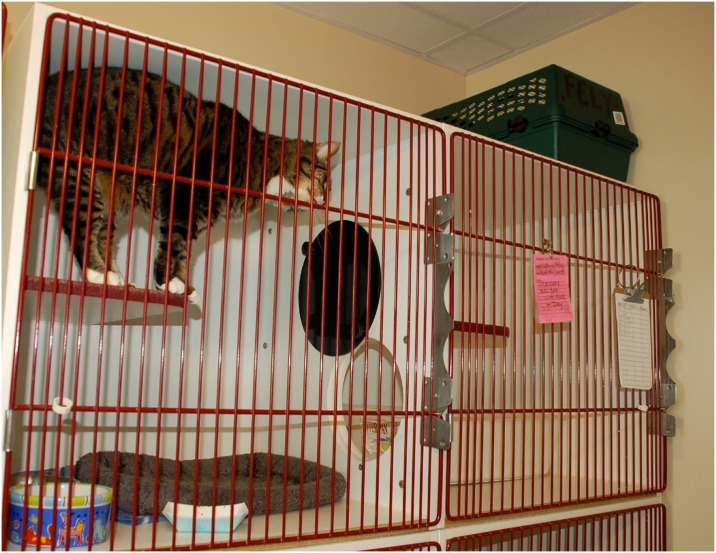
Feline housing ≥ 8–10 ft^2^ (0.74–0.93m^2^) of floor space and double compartment.

Cage floor space of >8 ft^2^ (0.74m^2^) was associated with significantly lower rates of URI compared to either cage floor space of < 6 ft^2^ (0.56m^2^) or floor space between 6–8 ft^2^ (0.56–0.74m^2^). There have been very limited studies on optimal single cat housing size for cats in confinement, so it is helpful to clarify the minimum size required to see a beneficial effect. Many “new and improved” kitty condos now on the market are in the 6–8 ft^2^ (.56-.74m^2^) range for floor space (~30–36” {76-91cm} long and 24–28” {61-71cm} deep) and may provide less benefit in terms of disease and stress reduction than larger models. Fig 2 shows a larger sized double-compartment housing unit.

Movement of cats in and out of the cage or between cages < 2 times in the first 7 days in the shelter was also significantly associated with lower risk of URI compared to more frequent movement. Change of housing has been associated with stress and activation of feline herpesvirus [15, 26].

Typical single-compartment cages often require daily removal or transfer of the cat for cleaning and care. Historically, this smaller single-compartment type housing has been commonly used in intake areas where cats spend their first 7 days in the shelter, while larger housing is reserved for cats in publicly accessible adoption areas. Replacing housing throughout the shelter with double-compartment cages or with larger walk-in units that facilitate cleaning with a minimum of disruption and handling for the cat may help reduce URI.

Separate housing for juveniles versus adults has been recommended as a strategy to control disease in shelters. Kittens are more susceptible to infection and shedding of respiratory pathogens [2, 11], which theoretically poses a risk to adult cats housed proximately. However, no significant effect was found on adult cat URI rates comparing shelters that house adults and juveniles separately versus in the same area. It may be that recrudescence of latent infection was more significant than transmission for adult cats in the shelters in this study. Therefore, the quality of housing and effects on stress reduction may be more significant factors in disease reduction than the separation of adult and kitten housing. From a shelter design and management perspective, housing adults and kittens in the same area provides more flexibility and potentially lowers costs by lowering the number of separate housing areas required. However, this study only analyzed URI rates in adults. It may be that separate housing would be beneficial for kittens, as it could theoretically protect them from adults subclinically shedding serious pathogens against which kittens have not yet developed immunity.

**Fig 3:**
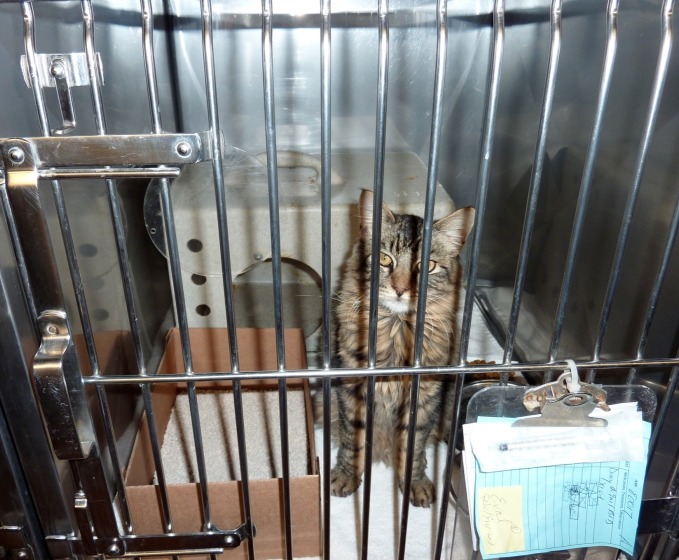
Single-cage housing unit 3-6ft^2^ (0.28–0.56m^2^) of floor space with solid hiding structure (feral den).

**Fig 4:**
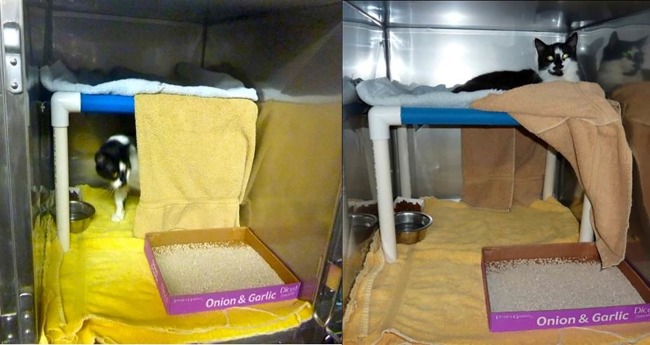
Single-cage housing unit 3-6ft^2^ (0.28–0.56m^2^) of floor space with a raised bed and a towel draped to provide hiding place with more usable floor space.

The association between provision of hiding places and *increased* URI rate was surprising, given studies documenting the benefit of hiding places for stress reduction in cats [27, 28]. Five of the six shelters that provided hiding places in this study housed cats in small cages (< 6 ft^2^ (0.56m^2^) of floor space). The addition of a solid hiding structure along with the litter box, food and water may have reduced available floor space to a level that negatively impacted cat health (Fig 3). Other ways of providing the ability to hide, such as a partial cage curtain, hanging a towel at the cage front, or draping a towel over a shelf or raised bed, may be preferable when smaller housing must be used (Fig 4).

Use of an intranasal URI vaccine was also significantly associated with a higher risk of URI. Although not expected to completely eliminate infection or illness, vaccination for FHV-1 and FCV is recommended to lower viral shedding and reduce clinical signs [29, 30]. Theoretically, intranasal vaccination should be particularly useful in a shelter environment where challenge may be encountered soon after admission, as it can elicit more rapid immunity than subcutaneous vaccination and can elicit a local mucosal immune response. One study showed a modest reduction in URI risk when a modified live intranasal FHV-1/FCV vaccine was used in conjunction with an inactivated subcutaneous vaccine, compared to the inactivated subcutaneous vaccine alone[19]. Another showed no significant benefit when a modified live intranasal FHV-1/FCV vaccine was used in conjunction with a modified live subcutaneous vaccine[18]. In clinical trials, intranasal vaccination has been associated with mild clinical signs in up to 30% of vaccinates. It could be that vaccine reactions appeared clinically identical to URI and were added to shelters reported URI rate, while vaccine failed to protect against more severe disease. Considering the increased risk found in this study, and the variable results of other studies, URI rates before and after use of an intranasal vaccine should be tracked to ensure no inadvertent adverse consequences and that the additional cost is justified.

Although it might be expected that control of URI is more challenging at larger shelters, intake number was not associated with any increased risk of URI amongst the shelters in this study. This is encouraging, as it suggests that URI can successfully be controlled at shelters spanning a range of sizes, not just at small shelters or those that strictly limit intake. However, the range of intake for shelters in this study was between ~ 700–4500 cats annually. It may be that greater differences would be found in much smaller or much larger shelters.

A concern for shelters may be that providing cats with larger, double-compartment housing will reduce the number of cats that could be housed at any one time. However, the number of cats housed in a shelter is a function of the number admitted and each cat’s length of stay (LOS). Enriched single or group housing environments have been associated with faster adoption and lower stress levels[31]. If improved housing also reduces illness, and therefore decreases length of stay, the same number of cats could potentially be served over time with fewer, larger housing units. Investment in flexible use housing, such as double-compartment cages with pass-through doors that can be closed between units, would allow shelters to track the impact of various housing configurations on cat health, length of stay, and the number of cats served. Structures such as stand-alone large pens or converted dog kennels can also be used for enriched, adequately sized single or group housing to provide additional flexibility[31, 32].

### Study limitations

Although each shelter used an internally consistent case definition for URI, a universal case definition could not be imposed. The definitions applied at each shelter formed a basis for management, housing and treatment decisions that varied across shelters. Case definitions ranged from inclusive (any single sign of URI such as sneezing, clear or mucopurulent nasal or ocular discharge) to strict (more than one sign of URI, mucopurulent nasal or ocular discharge and/or systemic illness). Theoretically, this could have led to overestimating the URI rate at the shelters with the most inclusive definitions, and underestimating at those shelters that had stricter standards for diagnosis. However, the shelter with the lowest URI rate also had the most inclusive definition, while the stricter definitions were found at shelters with higher URI rates. If anything, this would have led to a dampening of the differences in reported URI rates. The fact that significant differences were found despite this suggests the variations were the results of underlying environmental risks versus differences in case definition.

Another potential limitation of the study is that participating shelters inevitably experienced some changes in practices and circumstances over the course of the year that could affect URI rates. This likely occurred both unrelated to the study (e.g. staff turnover, weather events) and as a result of participation and learning more about URI (e.g., installation of larger cages, provision of hiding places). Shelters were asked to report any substantial changes monthly so that the effect of these changes could be accounted for when interpreting the results of the study. Changes in any of the analyzed factors were tracked and included in the final analysis. However, changes such as staff turnover reported by two of the shelters may have caused short term fluctuations in URI risk.

Additionally, other changes impacting URI rates may not have been recognized or reported. These could include impoundment of sick cats in association with cruelty or hoarding cases, fluctuations in the dog population affecting workload and feline care, or many other factors within the shelter. Voluntary reporting of possible variables was not sufficiently rigorous to support statistical analysis regarding the impact of these factors.

In addition to variation within each shelter, factors outside the shelter may have contributed to some of the observed variation in URI rates. Shelters spanned a wide range of climatic conditions from Canada to Southern California, and may have experienced weather or even socioeconomic factors affecting the risk of URI within the shelter.

Finally, some factors that might impact URI rate could not be evaluated. For instance, although airborne transmission is thought to be unlikely for feline URI, air quality likely has an impact on the risk for this disease [33, 34]. Air exchange rates, population density, and even cage size and design will all impact air quality at the level of a cat’s nose, and as such, would need to be specifically measured at each shelter and in each cage type. This type of detailed environmental analysis was beyond the scope of this study. Additionally, all the shelters shared some common practices: all shelters vaccinated cats on intake with a modified live parenteral vaccine for feline calicivirus, herpesvirus and panleukopenia; fed a consistent diet daily; and used a disinfection labeled as effective for all pathogens implicated in feline URI. Therefore, the impact of variations in these factors could not be evaluated.

In addition to physical risk factors, the role of handling, stress and behavioral health is increasingly appreciated as a significant factor affecting URI development in shelter cats. No shelter in the study reported a specific positive handling program such as has been described in association with decreased URI risk[3, 22, 23]. However, one shelter reported that larger, double compartment housing specifically facilitated minimal disruptive handling: following an intake exam and vaccination, cats were placed in the housing unit with food, water and bed in one compartment, a towel draped over the cage door to provide visual protection, and a litter box in the other compartment[35]. The cage was spot cleaned only as needed and food and water replaced without handling or removing the cat. It may be that some of the benefit of the larger cages at this shelter resulted from the reduction in disruptive handling versus the additional floor space per se.

This study provided a foundation to identify factors that may contribute to URI risk. Ideally, future research would build on this by prospectively evaluating the impact of changing a single factor, such as cage size or handling practices, and observing the URI rate in comparison with a control group of cats at the same shelter. Side-by-side comparisons, accompanied by detailed assessment including noise levels, temperature and air quality as well as behavioral stress scores for cats, would provide more specific information as to the relative importance of various environmental and management factors. As a supplement to formal research, routine monitoring of URI rates in shelters and reporting in context of possible contributing factors may help shed further light on practical methods to reduce this condition in shelter cats.

## Conclusions

URI is not an inevitable consequence of sheltering homeless cats. Housing and care of cats, particularly during their first week of stay in the shelter environment, significantly affects the rate of upper respiratory infection. Shelters can take immediate practical action based on the results of this study: investment in remodeling single units to double units that provide more space and are double compartment or purchase of larger housing units and provision of housing that does not require removing the cat for daily cleaning and care (double-compartment cage housing or housing that allows caretakers to enter the housing unit) will likely help reduce feline URI. As an added benefit, the type of housing associated with reduced URI risk may reflect lower stress levels for cats, and therefore may serve as an indicator that the shelter environment is more successfully meeting the cats’ needs for comfort and well-being.

## Supporting information

S1 Doc Shelter URI criteria. (DOCX)

S1 Survey link (DOCX)
