# Supplementary material for: Cage size, movement in and out of housing during daily care, and other environmental and population health risk factors for feline upper respiratory disease in nine North American animal shelters
Source: PLoS One. 2018 Jan 2;13(1):e0190140. doi: 10.1371/journal.pone.0190140 (PMC5749746; doi:10.1371/journal.pone.0190140)
Supplement: S1 Doc — (DOCX) [file pone.0190140.s001.docx]

Shelter URI Criteria

Shelter 1 - "mucoid nasal discharge, significant nasal congestion, fever, oral lesions, and/or inappetence all contribute to URI diagnosis. Cats with only ocular symptoms or cats with only sneezing and/or clear nasal discharge are not classified as URI."

Shelter 2 - All of the following are signs of feline upper respiratory disease. Any animal showing these signs should be isolated from the rest of the population even if no treatment is started. Clear or colored nasal discharge; Sneezing; Clear or colored discharge from one or both eyes; Red/inflamed conjunctiva (the pink tissue located around the eyes); Ulcers. Sores on the nose, lips, tongue or gums; Fever, lethargy, loss of appetite (can be sign of other disease as well)

Shelter 3 - Sneezing, purulent ocular and nasal discharge.

Shelter 4 - Nasal and/or ocular discharge; sneezing; congestion

Shelter 5 - The only time URI is diagnosed is by a veterinarian. In general the some (but certainly not all) of the following can constitute a dx of URI: moderate sneeze marks in cage more than 5d post IN vaccination, nasal discharge - serous or mucopurulent, ocular discharge - serous or mucopurulent, blepharospasm, corneal ulceration, conjunctivitis, chemosis, oral and nasal philtrum ulcers, congestion

Shelter 6 - Sneezing, depressed, etc. We have a URI grading chart the Veterinarians use.

Shelter 7 - Sneezing, clear or colored ocular and/or nasal discharge

Shelter 8 - Any symptoms of URI. We have a fairly strict definition of URI including Animal Care Workers observations

Shelter 9 – Sneezing and or purulent ocular or nasal discharge usually of 1 day of duration or longer
