# Supplementary material for: Cage size, movement in and out of housing during daily care, and other environmental and population health risk factors for feline upper respiratory disease in nine North American animal shelters
Source: PLoS One. 2018 Jan 2;13(1):e0190140. doi: 10.1371/journal.pone.0190140 (PMC5749746; doi:10.1371/journal.pone.0190140)
Supplement: S1 Survey link — (DOCX) [file pone.0190140.s002.docx]

S2 Survey link

Link to online Survey Results: <https://www.surveymonkey.com/results/SM-V52BFS8Z/>
